# Factors Associated With Postoperative Outcomes in Femtosecond Laser-Assisted Cataract Surgery

**DOI:** 10.7759/cureus.77933

**Published:** 2025-01-24

**Authors:** Nitin Deshpande, Vijay Shetty, Prajakta Desphande, Amruta Pradhan, Jekin Choubisa, Maninder S Setia

**Affiliations:** 1 Ophthalmology, Shree Ramkrishna Netralaya, Thane, IND; 2 Optometry, Shree Ramkrishna Netralaya, Thane, IND; 3 Epidemiology, Shree Ramkrishna Netralaya, Thane, IND

**Keywords:** cataract patients, eccentricity, femtosecond laser-assisted cataract surgery (flacs), postoperative outcomes, preoperative parameters

## Abstract

Background

This study aimed to assess the postoperative outcomes of patients who underwent femtosecond laser-assisted cataract surgery (FLACS) and the demographic and clinical factors associated with favorable visual outcomes.

Methodology

We conducted a secondary data analysis of 162 eyes from 145 patients at a tertiary eye hospital from January 2020 to August 2022. We collected pre and postoperative (one month) visual and refractive outcomes. We calculated the postoperative efficacy using the following formula: 1 - (postoperative cylinder - preoperative anticipated cylinder)/(preoperative cylinder - preoperative anticipated cylinder) × 100.

Results

The mean (SD) age of the study population was 64.9 (8.5) years. There was a significant change in the preoperative cylinder compared with the postoperative cylinder (-0.66 (-0.80, -0.56) D vs. 0 (-0.50, 0) D; p < 0.001). Postoperatively, about 69.8% (113) had an absolute cylinder of ≤0.25 D, 19.8% (32) had a cylinder of >0.25-0.50 D, and the remaining 10.5% (17) had a cylinder of >0.50 D. The median (interquartile range) preoperative cylinder was significantly different in the >0.50 D group compared with ≤0.25 D group (-0.80 (-1.00, -0.62) D vs. -0.65 (-0.75, -0.54) D; p < 0.001). The mean (SD) efficacy of the procedure was 72.2% (47.3%). The anterior eccentricity values at 8 mm chords were significantly different in the lower efficacy group and higher efficacy group (0.46 (0.29, 0.55) vs. 0.53 (0.42, 0.61); p = 0.04). The proportion of eyes with less than 100% efficacy was significantly higher in those with against-the-rule astigmatism compared with oblique and with-the-rule astigmatism (43.6% vs. 24.0%; p = 0.009).

Conclusions

The majority of the eyes operated with FLACS had postoperative astigmatism of less than or equal to 0.25 D and 100% postoperative efficacy, with no major complications. Lower efficacy of the procedure was associated with higher preoperative cylinder, length of the arc, lower eccentricity of the cornea, and against-the-rule astigmatism.

## Introduction

Femtosecond lasers work in the deep infrared zone, and the use of ultra-short pulses results in a perfect cut of tissues [1]. After the United States Food and Drug Administration approved ultrafast lasers more than two decades ago, femtosecond lasers have been used in refractive surgery, corneal surgery, and cataract surgery [1-5]. As this procedure does not require blades (as does conventional surgery), it may result in less trauma, endothelial cell loss, and faster healing in patients [1].

Since the initial use of femtosecond lasers in cataract surgery in 2008 [6], several studies have described the results of femtosecond laser-assisted cataract surgery (FLACS) or compared its outcomes with conventional cataract surgery, sometimes with different findings [7-9]. For instance, a study reported that the femtosecond arcuate keratotomy group had a higher correction index compared with conventional phacoemulsification [10]. Another study found that even though both the manual procedures and FLACS were safe, the latter had slightly superior outcomes and was precise and predictable [11]. Another retrospective study found that FLACS had better visual outcomes and fewer intraoperative complications compared with conventional phacoemulsification [12]. Other studies have reported that surgical-induced astigmatism was higher in the femtosecond group [13]. Systematic reviews and meta-analyses have also compared the outcomes of FLACS and other cataract procedures. A systematic review reported that both manual cataract surgery and FLACS were safe and moderately effective in correcting corneal astigmatism [14]. One meta-analysis found that FLACS did not improve intra or postoperative complications compared with the conventional phacoemulsification group [15]. However, another meta-analysis reported that complications such as posterior capsular tears were higher in the conventional phacoemulsification group [16]. In fact, some studies have reported that femtosecond laser-generated clear corneal incisions had lower endothelial gaping and misalignment [17].

The cost of the procedure may be an important factor in the use of this procedure in developing and underdeveloped countries. Indeed, some previous studies have suggested that FLACS may not be a cost-effective procedure [18,19]. Another important factor of concern is the length of surgical procedures due to additional procedure time in FLACS. Studies have highlighted that a significantly higher proportion of eyes achieved the target spherical equivalent of +0.50 D, with a visual acuity of 20/20, and low complications [20,21]. However, few studies have reported a detailed evaluation of the factors associated with better postoperative refraction and visual outcomes in patients undergoing this procedure. Thus, it is important to identify the factors associated with good postoperative outcomes in those undergoing the FLACS procedure for cataract surgery. This may help us identify individuals who are more likely to have good visual outcomes post-procedure.

With this background, we conducted the present analyses (1) to assess the postoperative refraction and vision in patients who underwent FLACS; (2) to evaluate the postoperative efficacy in these patients; and (3) to study the demographic and clinical factors associated with postoperative astigmatism and efficacy in these patients. The uniqueness of this study is that we have detailed factors associated with postoperative outcomes in FLACS. We examined the association between demographic factors (age, gender), cylinder (magnitude and axis), eccentricity, pachymetry, arc, and K1 and K2 with postoperative cylinder and postoperative efficacy.

## Materials and methods

The present study is a secondary data analysis of 162 eyes from 145 patients collected at a tertiary eye hospital from January 2020 to August 2022 in Thane, Maharashtra, India.

Study site and participants

Data were collected from patients attending a tertiary eye care center in Thane, Maharashtra, India. We included consecutive patients who had undergone FLACS-generated arcuate cuts. We included patients between the ages of 40 and 80 years with preoperative cylinder ranging from -0.50 to -1.50 D. We excluded patients who had conditions such as keratoconus, corneal ulcers, corneal opacities, previous ocular surgery, severe dry eyes, and post-LASIK patients.

Study procedure and variables

We abstracted the following data for the present analysis: (1) demographic data (age, gender); (2) operated eye; (3) preoperative cylinder and axis; (4) preoperative vision (corrected distance visual acuity (CDVA) measured using the logMAR chart and corrected near visual acuity (CNVA) measured with N notation chart); (5) corneal parameters such as K1 and K2 along with axis (the K1 and K2 measured in dioptres and axis in degrees using Zeiss IOL Master 700) and pachymetry (measured in µm using Zeiss Optical Coherence Tomography); (6) anterior and posterior eccentricity values at 4, 6, 8, and 10 mm chords (measured using Sirius Topography); (7) arc in the FLACS procedure (using the Woodcock nomogram); (8) postoperative vision (uncorrected distance visual acuity (UDVA), uncorrected near visual acuity (UNVA), CDVA, and CNVA (distance vision was measured using logMAR chart and near vision was measured using N notation chart)); and (9) postoperative sphere, cylinder, and axis (measured in dioptres and degrees). The postoperative outcomes were assessed at 28 ± 3 days (one-month outcome). We also calculated the postoperative efficacy using the following formula: 1 - (postoperative cylinder - preoperative anticipated cylinder)/(preoperative cylinder - preoperative anticipated cylinder) × 100.

Surgical procedure

All patients were operated on by a single surgeon. The FLACS machine was calibrated before the start of the procedure. We entered the patient’s demographic data and the type of procedure selected according to the choice of the intraocular lens (IOL). The machine scanned the eye through the imaging system and the pattern was created based on the density of cataracts as assessed by the scan. The various components in this procedure were (1) capsulotomy: the energy was set between 8 μJ and 20 μJ; spot separation varied between 4 μm and 10 μm, depending on the desired capsulotomy diameter; the diameter and centration were based on preoperative measurements and surgeon’s choice; the depth was usually set at around 500 μm for a clear lens capsule. (2) Lens fragmentation: the energy was set between 8 μJ and 20 μJ; the spot separation varied between 4 μm and 10 μm, depending on the desired fragmentation pattern; pattern selection: the surgeon could select potential patterns such as a cross, gird, or radial to customize the fragmentation of the cataractous lens. (3) Corneal incisions: the energy was set between 5 μJ and 15 μJ; the spot separation was set between 4 μm and 10 μm; and the depth was adjusted based on the required incision thickness and location. (4) Arcuate keratotomy: the energy was set between 5 μJ and 15 μJ; the spot separation was set between 4 μm and 10 μm; the length and depth of the arcuate incisions were determined based on the targeted astigmatism correction; precise arc-shaped incisions were created in the cornea to relax the steep meridian and flatten the cornea at the specific axis of astigmatism. After completion of the LENSAR process, we proceeded with phacoemulsification and IOL implantation.

Statistical analysis

We assessed the normality of linear variables using the Shapiro-Wilk test. We estimated the means and standard deviation (SDs) for normally distributed data. We estimated the medians and interquartile range (IQR) for data that were not normally distributed. As we had correlated data (multiple eyes of the same patients), we used linear mixed models to compare the differences between groups. These models are useful for correlated data in ophthalmologic studies, as standard methods (such as analysis of variance (ANOVA)) may overestimate the effect [22,23]. We adjusted for patient-level data in these models and the models were coded according to the number of eyes and the number of patients. We built individual models for each variable to examine the association between clinical parameters and the postoperative cylinder. We also built individual models to examine the association between clinical parameters and post-operative efficacy. Initially, we assessed the fit of these models compared with simple regression models using likelihood ratio tests. After this, the fit of the linear mixed models was assessed using the Akaike information criteria and Bayesian information criteria [24-26]. We estimated the proportions for categorical variables. The proportions were compared using the chi-square test or Fisher’s exact test for low expected cell counts. For the categorical comparison, we used the degrees of freedom for the appropriate row X column. For the 2 × 2 chi-square test, the degree of freedom was 1; for the 2 × 3 or 3 × 2 chi-square test (such as gender [2] and postoperative cylinder groups [3], or type of astigmatism [3] and postoperative efficacy [2]), the degrees of freedom were 2; and for 3 × 3 chi-square test (such as type of astigmatism [3] and postoperative cylinder groups [3]), the degrees of freedom were 4. A p-value <0.05 was considered statistically significant. Data were entered in MS Excel (Microsoft Corp., Redmond, WA, USA) and analyzed using Stata Version 17 (StataCorp., College Station, TX, USA).

Ethical considerations

The study was approved by the Institutional Ethics Committee of Shree Ramkrishna Netralaya (approval number: SRNEC/ECD/2022/001; dated December 05, 2022).

## Results

The mean (SD) age of the study population was 64.9 (8.5) years. About 46% (67) were males and 54% (78) were females. The median (IQR) preoperative cylinder was -0.66 (-0.80, -0.56) D and the median (IQR) axis was 80.5 ° (29.0°, 125.0°). The mean (SD) K1 and K2 values were 43.4 (1.4) D and 44.1 (1.5) D, respectively, and the mean corneal thickness on pachymetry was 540.8 (36.7) µm. The mean (SD) arc size during the procedure was 25.7° (9.6°). The median (IQR) postoperative spherical value was 0 (0, 0) D, the cylinder was 0 (-0.50, 0) D, and the spherical equivalent was 0 (-0.125, 0) D. The median (IQR) add value was 2.50 (2.25, 2.50) D postoperatively. There was a significant change in the postoperative cylinder compared with the preoperative cylinder (p < 0.001). We have presented these values in Table 1. The median (IQR) CDVA was 0 (0, 0) logMAR units and UDVA was 0 (0, 0.14) logMAR postoperatively. About 27.0% had UNVA of N6, 19.5% had N8/N10, 38.9% had N12/N18, and 14.5% was less than N18. However, 96.8% had CNVA was N6, 1.3% had N8/N10, and 1.9% had less than N18.

**Table 1:** Preoperative and postoperative cylinder in in 162 eyes undergoing femtosecond laser-assisted cataract surgery. There was a significant change in the postoperative and preoperative cylinder (p < 0.001). P-values were calculated using linear mixed models for correlated data.

Cylinder (magnitude) in D	Preoperative	Postoperative
Median	-0.66	0.0
Interquartile range	-0.80, -0.56	-0.50, 0.0

**Figure 1:**
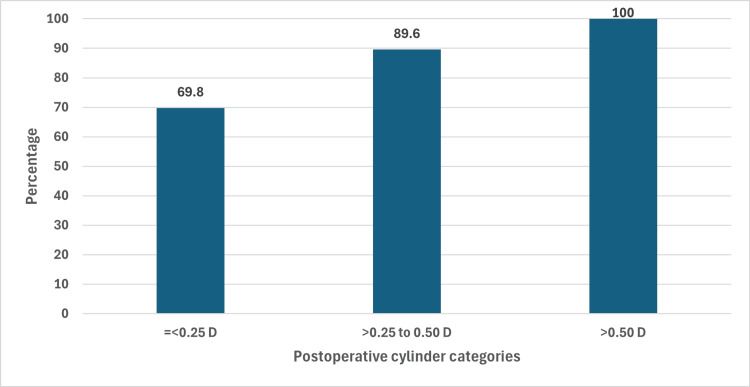
Bar chart of absolute values of postoperative cylinder categories in 162 eyes that underwent femtosecond laser-assisted cataract surgery. The X-axis is the categories of the absolute value of the postoperative cylinder and the Y-axis is the percentage. Each bar is a cumulative percentage till that group.

**Figure 2:**
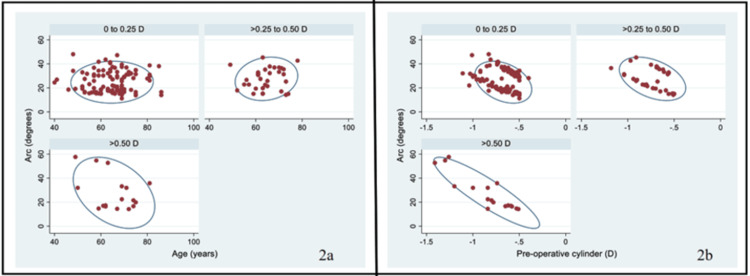
Scatter plot showing the distribution of age (a) and preoperative cylinder (b) on X-axis and arc on Y-axis. A confidence ellipse has also been plotted according to the three categories of postoperative cylinder (0 to 0.25, >0.25 to 0.50, and >0.50) in 162 eyes that underwent femtosecond laser-assisted cataract surgery. In the 0 to 0.25 D and >0.25 to 0.50 D groups, the values were clustered; however, there was variability in the >0.50 D group.

Post-operatively, about 69.8% (113) had an absolute cylinder of ≤0.25 D, 19.8% (32) had a cylinder of >0.25 to 0.50 D, and the remaining 10.5% (17) had a cylinder of >0.50 D. We have presented cumulative proportions of postoperative absolute cylinder in Figure 1. The proportion of eyes with the absolute cylinder of >0.50 D was higher in those above the age of 70 years compared with those under 60 years of age; however, the difference was not statistically significant (13.9% vs. 9.2%; p = 0.39). The median (IQR) preoperative cylinder was not significantly different in the >0.25 to 0.50 D group compared with the ≤0.25 D group (-0.69 (-0.87, -0.58) D vs. -0.65 (-0.75, -0.54) D; p = 0.079). However, the median (IQR) preoperative cylinder was significantly different in the >0.50 D group compared with ≤0.25 D group (-0.80 (-1.00, -0.62) D vs. -0.65 (-0.75, -0.54) D; p < 0.001). Finally, the median (IQR) was also significantly different in the >0.50 D group compared with the >0.25 to 0.50 D group (-0.80 (-1.00, -0.62) D vs. -0.69 (-0.87, -0.58) D; p = 0.025). We also found that the mean (SD) arc was higher in the >0.50 D group compared with the ≤0.25 D group (27.8° (14.8°) vs. 24.8° (8.7°); p = 0.22), but the difference was not statistically significant. We also plotted the arc and preoperative cylinder according to the categories of postoperative cylinder. As seen in Figure 2, in the 0 to 0.25 D and >0.25 to 0.50 D groups, the values were clustered; however, there was variability in the >0.50 D group. The proportion of eyes with postoperative cylinder ≤0.25 D was higher in with-the-rule (74.6%) and oblique (80.0%) astigmatism compared with against-the-rule astigmatism (58.1%) (p = 0.08). Detailed analyses have been presented in Table 2.

**Table 2:** Demographic and clinical factors associated with postoperative cylinder in 162 eyes that underwent femtosecond laser-assisted cataract surgery. ^a^: 0 to 0.25 vs. >0.25 to 0.50; p < 0.05; ^b^: 0 to 0.25 vs. >0.50; p < 0.05; ^c^: >0.25 to 0.50 vs. >0.50; p < 0.05. Test statistics: Linear mixed models for correlated data; proportions were compared using the chi square test. The appropriate chi-square test based on the number of rows and columns was used for categorical data. IQR: interquartile range; SD: standard deviation; AIC: Akaike information criterion; BIC: Bayesian information criterion

Parameters	Total	0 to 0.25 D	> 0.25 to 0.50 D	> 0.50 D	P-value	AIC	BIC
	N (%)	n (%)	n (%)	n (%)			
All	162 (100)	113 (69.8)	32 (19.8)	17 (10.5)			
Age (years)							
40–59	40 (24.7)	30 (75.0)	6 (15.0)	4 (10.0)	0.75	-	-
60–69	79 (48.8)	56 (70.9)	16 (20.3)	7 (8.9)			
≥70	43 (26.5)	27 (62.8)	10 (23.3)	6 (13.9)			
Gender							
Male	74 (45.7)	56 (75.7)	12 (16.2)	6 (8.1)	0.32	-	-
Female	88 (54.3)	57 (64.8)	20 (22.7)	11 (12.5)			
Eye							
Right eye	81 (50.0)	50 (61.7)	19 (23.5)	12 (14.8)	0.06	-	-
Left eye	81 (50.0)	63 (77.8)	13 (16.1)	5 (6.2)			
Preoperative cylinder, median (IQR)	-0.66 (-0.80, -0.56)	-0.65 (-0.75, -0.54)	-0.69 (-0.87, -0.58)	-0.80 (-1.0, -0.62)	0.0002^b, c^	-99.05	-83.61
Arc (degrees), mean (SD)	25.7 (9.6)	24.8 (8.7)	27.6 (9.1)	27.8 (14.8)	0.21	1,198.22	1,213.66
K1, mean (SD)	43.4 (1.4)	43.3 (1.4)	43.7 (1.5)	43.8 (1.9)	0.76	308.30	320.74
K2, mean (SD)	44.1 (1.5)	43.9 (1.4)	44.4 (1.5)	44.6 (2.0)	0.45	311.09	323.54
Pachymetry, mean (SD)	540.8 (36.7)	538.0 (35.1)	549.9 (36.1)	543.8 (48.9)	0.44	1,030.92	1,044.10
Eccentricity, mean (SD)							
Anterior @ 4	0.44 (0.26, 0.57)	0.46 (0.29, 0.57)	0.44 (0.35, 0.64)	0.22 (-0.34, 0.38)	0.016^b, c^	62.98	76.10
Anterior @ 6	0.39 (0.26, 0.50)	0.41 (0.32, 0.51)	0.33 (-0.09, 0.42)	0.34 (0.24, 0.50)	0.07^a^	24.39	37.51
Anterior @ 8	0.50 (0.39, 0.60)	0.53 (0.42, 0.61)	0.39 (0.18, 0.53)	0.54 (0.40, 0.64)	0.008^a, c^	-50.40	-37.32
Anterior @ 10	0.67 (0.58, 0.73)	0.68 (0.60, 0.72)	0.63 (0.54, 0.68)	0.71 (0.67, 0.77)	0.26^c^	-114.36	-101.69
Type of cylinder							
With the rule	55 (33.9)	41 (74.6)	11 (20.0)	3 (5.5)	0.08	-	-
Oblique	45 (27.8)	36 (80.0)	6 (13.3)	3 (6.7)			
Against the rule	62 (38.3)	36 (58.1)	15 (24.2)	11 (17.7)			

**Figure 3:**
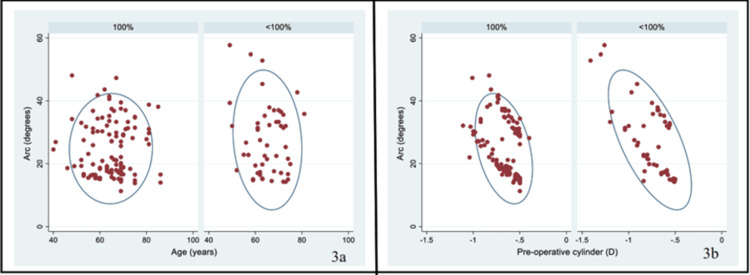
Scatter plot showing the distribution of age (a) and preoperative cylinder (b) on the X-axis and arc on the Y-axis. A confidence ellipse has also been plotted according to efficacy (100% and <100%) in 162 eyes that underwent femtosecond laser-assisted cataract surgery. The values of preoperative cylinder and arc appear to be closely clustered in the group with 100% efficacy. There was a lot of variability in the other group with an efficacy of <100% efficacy.

The mean (SD) efficacy of the procedure was 72.2% (47.3%) and the median (IQR) efficacy was 100% (42.3%, 100%). About 68.5% (111) had 100% efficacy, and the remaining 31.5% (51) had less than 100% efficacy. On comparing the optic parameters between these two groups, we found that the median (IQR) preoperative cylinder was significantly different in the <100% efficacy group compared with the 100% efficacy group (-0.73 (-0.90, -0.59) D vs. -0.65 (-0.75, -0.54) D; p = 0.0007). The mean (SD) arc was higher in those who had lower efficacy (27.6 (11.1) vs. 24.8 (8.8); p = 0.09). Although, in general, the anterior eccentricity values were low in the low efficacy group, it was significantly different at 8 mm chords (0.46 (0.29, 0.55) vs. 0.53 (0.42, 0.61); p = 0.04). The proportion of eyes with less than 100% efficacy was significantly higher in those with against-the-rule astigmatism compared with oblique and with-the-rule astigmatism (43.6% vs. 24.0%; p = 0.009). We have presented detailed data in Table 3. As shown in Figure 3, the values of preoperative cylinder and arc appear to be closely clustered in the group with 100% efficacy; there was considerable variability in the other group with an efficacy of <100% efficacy. None of the eyes reported any complication at one month post-surgery.

**Table 3:** Clinical factors associated with postoperative efficacy in 162 eyes that underwent femtosecond laser-assisted cataract surgery. ^a^: 0 to 0.25 vs. >0.25 to 0.50; p < 0.05; ^b^: 0 to 0.25 vs. >0.50; p < 0.05; ^c^: >0.25 to 0.50 vs. >0.50; p < 0.05. Test statistics: Linear mixed models for correlated data; proportions were compared using the chi square test. The appropriate chi-square test based on the number of rows and columns was used for categorical data. IQR: interquartile range; SD: standard deviation; AIC: Akaike information criterion; BIC: Bayesian information criterion

Parameters	Total	Efficacy (100%)	Efficacy (<100%)	P-value	AIC	BIC
All, n (%)	162 (100)	111 (68.5)	51 (31.5)			
Preoperative cylinder, median (IQR)	-0.66 (-0.80, -0.56)	-0.65 (-0.75, -0.54)	-0.73 (-0.90, -0.59)	0.0007	-95.79	-83.44
Arc (degrees), mean (SD)	25.7 (0.76)	24.8 (8.8)	27.6 (11.1)	0.09	1196.40	1208.75
K1, mean (SD)	43.4 (1.4)	43.3 (1.4)	43.7 (1.6)	0.50	306.39	316.35
K2, mean (SD)	44.1 (1.5)	43.9 (1.4)	44.4 (1.6)	0.21	309.16	319.12
Pachymetry, mean (SD)	540.8 (36.7)	538.2 (35.4)	547.2 (39.7)	0.23	1,029.16	1,039.70
Eccentricity, mean (SD)						
Anterior @ 4	0.44 (0.26, 0.57)	0.46 (0.28, 0.57)	0.38 (0.23, 0.64)	0.27	68.10	78.60
Anterior @ 6	0.39 (0.26, 0.50)	0.41 (0.32, 0.51)	0.33 (0.17, 0.47)	0.13	25.07	35.57
Anterior @ 8	0.50 (0.39, 0.60)	0.53 (0.42, 0.61)	0.46 (0.29, 0.55)	0.04	-46.20	-35.74
Anterior @ 10	0.67 (0.58, 0.73)	0.68 (0.60, 0.72)	0.67 (0.55, 0.74)	0.44	-114.27	-104.14
Type of cylinder, n (%)						
With the rule	55 (33.9)	41 (74.6)	14 (25.5)	0.032	-	-
Oblique	45 (27.8)	35 (77.8)	10 (22.2)			
Against the rule	62 (38.3)	35 (56.5)	27 (43.6)			

## Discussion

We found that a high proportion of eyes that had undergone cataract surgery with the FLACS procedure had a postoperative cylinder of 0 to 0.25 D (absolute values), and the efficacy of the procedure was 100% in the majority of them. Eyes that had a postoperative cylinder of >0.50 D had a significantly higher median magnitude of the preoperative cylinder and against-the-rule astigmatism. The factors associated with less than 100% postoperative efficacy were higher preoperative cylinder magnitude, lower anterior eccentricity of the cornea assessed at 8 mm chords, and against-the-rule astigmatism.

FLACS is considered a precise procedure for cataract surgery and reduces the risk associated with the human component of the surgery [27]. Thus, it is possible to standardize the surgical procedure and improve postoperative visual outcomes. It has also been reported that along with better visual outcomes (such as UDVA and CDVA), the FLACS group had significantly lower cumulative dissipated energy compared with the conventional group [12]. However, there may be a learning curve associated with this procedure. For instance, Christy et al. reported that about 25 to 30 cases may be required for reproducible results with FLACS [28]. Zhang et al. reported that 100 cases were required for the basic learning curve and 150 cases may be required for the advanced learning curve [29]. Another study by Roberts et al. suggested that complications such as anterior capsular tear and posterior capsular rupture may be common in the initial 14 to 16 cases of FLACS [30].

As seen in our study, Ang et al. also reported that the refractive and visual outcomes had improved in those who underwent the FLACS procedure along with lower postoperative anterior chamber inflammation and endothelial cell loss [31]. Their mean (SD) cylinder was -1.23 (0.82) D, which changed to -0.70 (0.40) D. They also found that the mean (SD) CDVA had changed from 0.25 (0.35) D preoperatively to 0.03 (0.08) D. However, they found 66% of the eyes had a postoperative cylinder of <0.50 D. [31] Another study by Day et al. reported that mean (SD) UDVA postoperatively was 0.13 (0.22) logMAR units, and 71% of the eyes had mean spherical refraction within +0.50 D. [19] In our study, the median postoperative UDVA and CDVA was 0 logMAR units. Furthermore, in our study, 70% of the eyes had a postoperative cylinder of ≤0.25 D, and 89.5% had a postoperative cylinder of ≤0.50 D. Thus, we had slightly better results compared with these previous studies. Both the above-mentioned studies did not assess the factors associated with good postoperative refraction in patients who underwent FLACS surgery. This was evaluated in our study. We found that a lower postoperative cylinder (≤0.25 D) was associated with preoperative cylinder, eccentricity, and type of astigmatism. Other studies have reported that visual and refractive outcomes had little difference between FLACS and other procedures [32-34]. Shaheen et al. also did not find any correlation between axial length or preoperative spherical equivalent and postoperative mean absolute error [11]. In our study, we found that there was an association between the preoperative cylinder values, as well as postoperative cylinder and efficacy. Previous studies have reported complications such as anterior capsular tears, incomplete capsulotomies, suction breaks, endophthalmitis, and miosis associated with this procedure [29,35-38]. Some common complications reported by Nagy et al. included miosis (32%), capsular tears and bridges (20%), and anterior tears (4%) [36]. However, we found that the procedure was safe, and we did not encounter any complications in these patients.

The factors associated with poor efficacy in our study were higher preoperative cylinder and lower anterior eccentricity. Previous reports have indicated that poor refractive outcomes in FLACS surgery were associated with previous surgery and poorer preoperative visual acuity [39]. Corneal eccentricity essentially estimates the flattening of the peripheral radius of the cornea compared with the apical radius [40]. Reeves proposed that mean eccentricity is higher toward the periphery of the cornea [40]. Other studies have found that eccentricity is associated with age, refractive error, and anterior segment volume and angle [41]. A recent study found that corneal curvature is positively correlated with anterior corneal eccentricity [42]. Thus, these factors may also be considered in the algorithms of FLACS. Park et al. proposed that higher postoperative astigmatism was associated with higher preoperative astigmatism and higher eccentricity [43]. In our study, we found that a higher postoperative astigmatism was associated with a higher preoperative astigmatism. The direction of astigmatism also affected the efficacy in our study; it was lower in those who had against-the-rule astigmatism. Lowry and colleagues, however, found that overcorrection was observed in patients who had with-the-rule astigmatism [44]. In fact, they suggested that the axis of astigmatism should also be considered for lens selection and adjustments may be made for over or under-correction [44].

There may be some practical issues for the use of FLACS. For instance, Bénard et al. reported that the incremental cost-effectiveness ratio of FLACS was not considered with the amount that may be classified as cost-effective [45]. Another study by George et al. found the return on investment to be rapid in a rural ophthalmology setting [46]. Day and colleagues, however, reported that FLACS was not a cost-effective procedure [47]. Another issue associated with the use of FLACS in surgical settings is the time required for some of the added steps during surgery. Indeed, Lubahn et al. reported additional time of 11.6 to 13.4 minutes in routine surgeries that used the FLACS procedure [48]. Thus, the cost of the procedure and increased time during surgery may have to be considered particularly in low-resource and high-volume settings.

Strengths and limitations

The study had some limitations. For instance, we did not have any comparison group. Many previous studies have compared the FLACS procedure with conventional phacoemulsification procedures. However, we were more interested in understanding the issues that were associated with postoperative outcomes in eyes that underwent cataract surgery with the FLACS procedure. Furthermore, we have not included certain other outcomes, such as cumulative dissipated energy in our analysis. As these were secondary data, we did not have information on this variable. However, the study had some strengths. We used linear mixed models for the analysis of our data. These models are better for correlated data as traditional methods (such as ANOVA) overestimate the significance. Although many previous studies have reported postoperative vision and refraction, they have not studied/reported factors associated with these outcomes. In our study, we have added to this component of the literature by examining the factors associated with postoperative refraction and efficacy in these patients.

## Conclusions

Despite these limitations, the study provides useful information on the factors associated with visual and refractive outcomes in FLACS. We found that, overall, the postoperative cylinder was ≤0.25 D in nearly 70% of the eyes with nearly 90% of eyes with a cylinder of ≤0.50 D. The median UDVA and CDVA were 0 logMAR units for both. In our study, lower efficacy of the procedure was associated with higher preoperative cylinder, length of the arc, lower eccentricity of the cornea, and against-the-rule astigmatism. Thus, overall, we found that the procedure had good postoperative refractive and visual outcomes, and was safe without any major complications. Based on our findings, we suggest that the eccentricity of the cornea and the direction of the astigmatism should also be included to modify the algorithm. This may improve postoperative efficacy and refractory outcomes in these patients.
